# Detection of HPV DNA in Cervical Intraepithelial Neoplasia Using In Situ Hybridization

**DOI:** 10.3390/jcm15103974

**Published:** 2026-05-21

**Authors:** Marcin Przybylski, Sonja Millert-Kalińska, Dominik Pruski, Mateusz de Mezer, Monika Krzyżaniak, Robert Jach, Jakub Żurawski, Paweł Kurzawa

**Affiliations:** 1 Department of Obstetrics and Gynecology, District Public Hospital, 60-479 Poznań, Poland; dominik.pruski@icloud.com; 2 Department of Immunobiology, Poznan University of Medical Sciences, 60-812 Poznań, Poland; 3 Department of Oncological Pathomorphology, University Clinical Hospital in Poznan, 60-572 Poznań, Poland; 4 Department of Gynecological Endocrinology, Jagiellonian University Medical College, 31-008 Kraków, Poland

**Keywords:** FFPE, ISH, HPV DNA, CIN, SIL, in situ hybridization, pathomorphology

## Abstract

**Background**: Human papillomavirus (HPV)-related diseases remain a major global health problem, with cervical intraepithelial neoplasia (CIN) representing a key precursor to cervical cancer. Identification of high-risk HPV genotypes is essential for early diagnosis and appropriate management. This study aimed to evaluate the usefulness of in situ hybridization (ISH) for detecting HPV DNA in formalin-fixed, paraffin-embedded (FFPE) cervical tissue and to compare automated signal detection with manual histopathological assessment. **Methods**: This prospective, non-randomized study included 83 women undergoing diagnostic procedures for abnormal cytology or confirmed CIN between 2022 and 2023. Tissue specimens obtained during a loop electrosurgical excision procedure (LEEP) were examined using two ISH probes: ISH II for low-risk HPV types 6 and 11, and ISH III for high-risk HPV genotypes. Staining patterns and distributions were evaluated and correlated with molecular HPV testing and histopathological outcomes. **Results**: ISH II distribution was significantly associated with the presence of HPV type 6 or 11 (*p* < 0.001), although stain structure itself was not. ISH III stain structure was significantly associated with high-risk HPV genotypes (*p* = 0.020). A positive ISH II result predicted low-risk HPV infection with a sensitivity of 62.5% and specificity of 64.0%, while ISH III predicted high-risk HPV infection with a sensitivity of 86.36% but lower specificity (23.53%). Overall diagnostic accuracy was 63.86% for ISH II and 73.49% for ISH III. **Conclusions**: ISH proved to be a reproducible method for detecting HPV in archived cervical tissue, enabling assessment even years after specimen collection. Although PCR-based methods remain more widely used due to higher sensitivity and less invasive sampling, ISH provides valuable morphological context and may serve as a complementary diagnostic tool, particularly when only archival tissue is available.

## 1. Introduction

HPV-related diseases remain a significant health problem worldwide. However, cervical cancer incidence and mortality rates have declined significantly in high-income countries in Europe, North America, and Australia in recent decades. Cervical cancer (cc) is the second most common cancer in women worldwide, with an estimated 529,000 new cases and 275,000 deaths annually [1]. The implementation of population-wide HPV vaccination of girls before sexual initiation a few decades ago resulted in the complete disappearance of cervical cancer in the youngest age groups of women in Australia and according to WHO it might be one of first countries to eliminate cc [2]. The use of HPV vaccination in the primarily unvaccinated population, including adolescent and adult women, also has a beneficial effect on eliminating infection in the adult population [3,4,5]. A growing body of literature supports this association, but further follow-ups of 10–20 years and larger cohorts are needed to strengthen this evidence.

For now, however, the cervix and precancerous conditions in the head and neck, vagina, vulva, penis, and cervix constitute a significant global health problem. For this reason, scientists are searching for the most sensitive and accessible methods for detecting early HPV-related lesions. Identifying patients in high-risk groups will allow for early diagnostic and therapeutic interventions. This applies to various groups of physicians—from gynecologists and proctologists to head and neck surgeons and otolaryngologists. Therefore, diagnostic tools effective in the prevention and treatment of cervical intraepithelial neoplasia and cervical cancer will impact the diagnosis and treatment of lesions in other locations to which the HPV virus also has a tropism.

The link between infection with a highly oncogenic human papillomavirus genotype and the development of cervical neoplasia was already demonstrated in the 20th century by Harald zur Hausen [6]. HPV is a double-stranded DNA virus with over 200 genotypes and approximately 7900 base pairs. The HPV life cycle is directly linked to keratinocyte differentiation. One of the key events in the virus life cycle is the escalation of viral replication, dependent on keratinocyte differentiation. This increase in replication activity leads to amplification of the HPV genome, from approximately 50 copies per cell in basal keratinocytes during the subclinical phase of infection to thousands of copies per cell in suprabasal keratinocytes during the clinical phase of infection. HPV infection of the cervical epithelium has been recognized as a factor initiating the oncogenic process leading to the development of cervical cancer [7]. The risk of disease progression from early precancerous stages to cervical cancer depends on the HPV type. Therefore, HPV genotypes have been grouped into different oncogenic risk groups. Highly oncogenic genotypes are HPV 16, 18, 31, 33, 35, 39, 45, 51, 52, 56, 58, 59, 66, and 68 and have a significant potential to cause precancerous conditions [8]. Identifying infection with a highly oncogenic HPV type is currently the most important factor in identifying high-risk patients. The accumulating mucus in the ecto- and endocervix is an ideal site for collecting material for HPV DNA testing. Molecular testing at this site is characterized by the highest diagnostic value and the highest reproducibility, rarely seen in medicine. Not every anatomical site can be used for a traditional swab for molecular testing for HR HPV. Therefore, the world is searching for better methods to confirm HPV infection from biopsy material from the vagina, vulva, anus, penis, or nasopharynx. This study aims to assess whether hybridization techniques can detect specific HPV types during histopathological evaluation of tissue specimens.

Identifying HPV risk groups using in situ hybridization (ISH) on slides, combined with conventional slide morphology assessment, can improve clinical decision-making. ISH on slides is an easy-to-perform, reproducible method for detecting HPV in paraffin-embedded tissue sections. Detection levels for both radioisotopic and non-isotopic markers using genomic probes appear low in formalin-fixed tissue samples; the number of viral copies per cell may be as low as 10–50. An additional advantage of testing from paraffin blocks is the ability to perform testing several months or years after the procedure, when it is no longer possible to collect a smear for molecular HPV testing. In the event of recurrence of intraepithelial neoplasia or anogenital cancer, testing can be performed from archival material such as paraffin blocks. Another way to interpret ISH is semi-quantitative analysis of immunopositive cells performed based on the images from the light microscope. Phase analysis of immunohistochemically stained slides is used, including automatic detection of objects based on their color, shade intensity or shape [9].

The following work compares the results of automated detection of the luminous signal in images using ISH II and ISH III probes with manual assessment by a pathologist. Future development of AI techniques will likely significantly influence the further development of this type of technique. Computer analysis of cytological slides to support cytodiagnosticians and pathologists is already being implemented in developed countries; however, such solutions should be supervised by a human operator.

## 2. Materials and Methods

### 2.1. Study Design

We provide a prospective, ongoing 12-month, non-randomized study in patients reporting to the Individual Specialized Medical Practice and District Public Hospital in Poznan, Poland, between 2022 and 2023. Subjects attended the medical practice as part of in-depth diagnostics due to an abnormal cytological result or the presence of HPV 16, 18, or 31 in the cervical smear or because of histopathologically confirmed cervical intraepithelial neoplasia. All patients requiring treatment of precancerous HSILs (CIN 2+) underwent a LEEP in accordance with the recommendations of the Polish Society of Obstetricians and Gynecologists. The histopathologist re-verified the diagnosis and marked the places of CIN lesions on theformalin-fixed, paraffin-embedded (FFPE) cervical tissues. The laboratory diagnostician prepared new slide preparations for ISH staining. In two cases, the final medical consensus did not confirm the diagnosis of CIN; therefore, they were excluded from further analysis. The Poznan University of Medical Sciences Bioethical Committee approved the study protocol (540/22) on Jun 23 2022. The present study uses the same patient cohort as our previous publication evaluating automated ISH signal detection; however, the current analysis focuses on conventional microscopic interpretation by a pathologist rather than computer-assisted image analysis.

### 2.2. HPV Genotyping Test and LBC

We collected liquid-based cytology and molecular assessment samples using an endocervical Cyto-Brush, which were preserved in BD SurePath^®^ (Becton, Dickinson and Company, Franklin Lakes, NY, USA). Then, the probes were passed to an independent, standardized laboratory. PCR was performed, followed by a DNA enzyme immunoassay and HPV genotyping using a reverse hybridization line probe assay. The lab technicians performed sequence analysis to characterize HPV-positive samples. The molecular test detected the DNA of 37 HPV genotypes (6, 11, 16, 18, 26, 31, 33, 35, 39, 40, 42, 45, 51, 52, 53, 54, 55, 56, 58, 59, 61, 62, 64, 66, 67, 68, 69, 70, 71, 72, 73, 81, 82, 83, 84, IS39, and CP6108).

### 2.3. Colposcopy and Punch Biopsy

Further validation of abnormal screening results was performed on all patients with an abnormal smear: ASC-US, LSIL, HSIL, ASC-H, AGC, cervical cancer, and a positive HPV test for types 16, 18, and 31, and a clinically suspicious cervical image as described in a previous study [10].

### 2.4. Immunohistochemistry

Serial 4-micrometer tissue sections were cut from the donor blocks containing cores of lesions and applied to special immunohistochemistry-coated slides. Two ISH probes—INFORM HPV II Family 6 Probe and INFORMHPV III Family 16 Probe (Ventana, Roche, Tucson, AZ, USA)—were used to target the common HPV genotypes in cervical biopsy specimens. To demonstrate positive hybridization to low-risk genotypes 6 and 11, the INFORM HPV II Family 6 Probe and the INFORMHPV III Family 16 Probe were used to demonstrate positive hybridization to the following genotypes: 16, 18, 31, 33, 35, 45, 52, 56, 58, and 66. Slides were stained on a fully automated immunohistochemistry slide stainer, Bench-Mark ULTRA (Ventana, Roche, Tucson, AZ, USA). Staining protocol parameters were based on HIER using CC2 (heating time 4 + 8 + 8 min at 86 °C), ISH-Protease 3 (780-4149) for 16 min, 12 min of denaturation, and 2 h of hybridization with each ISH probe. To detect specific DNP-labeled probes bound to a target sequence, an indirect biotin–streptavidin system (INFORM iView Blue +, 760-097) was used. Slides were then post-counterstained with Red Stain II (780-2218) (Ventana, Roche, Tucson, AZ, USA) for 4 min. Coverslips were passed through a series of alcohols and finally xylene before being mounted. The complex, visualized with the chromogen 5-bromo-4-chloro-3-indolylphosphate (BCIP) and nitroblue tetrazolium (NBT), forms a blue precipitate that is easily detected under a light microscope.

### 2.5. Histopathological Evaluation of the Preparations After ISH Examination

The slides were repeatedly evaluated by a pathologist specializing in gynecologic oncology in the presence of two gynecologists, including one gynecologic oncologist, who jointly confirmed the presence of staining and its nature as defined. The following features were assessed jointly:

- Whether there is staining in ISH II or ISH III;
- Whether there is a diffused/punct/negative stain;
- Whether there is a distribution of the focal/dispersed/negative type.

**Figure 1:**
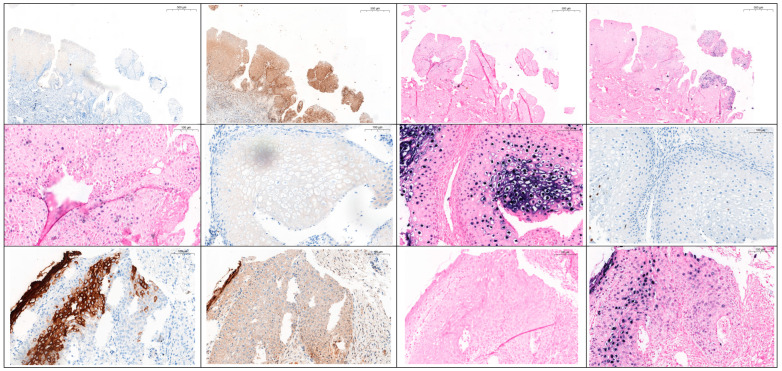
Representative cervical tissue specimens stained using different HPV-related markers and detection methods, including E4 immunohistochemistry (E4 IHC), pan-HPV immunohistochemistry (HPV IHC), HPV 6 in situ hybridization (HPV 6 ISH), and HPV 16 in situ hybridization (HPV 16 ISH). Patient 1–28 years old, LBC LSIL, HPV 6, 16, 31, CIN 3, p16/Ki67 (+), picture 1—staining E4, picture 2—staining HPV, picture 3—staining ISH II, picture 4—staining ISH III, picture 5—staining ISH II. Patient 2—25 years old, LBC LSIL, HPV 6, CIN 2/3, p16/Ki67 (+), picture 6—staining E4, picture 7—staining ISH II. Patient 3—45 years old, LBC ASC-H, HPV 18, 39, CIN 2/3, p16/Ki67 (+), picture 8—staining HPV, picture 9—staining E4, picture 10—staining HPV, picture 11—staining ISH II, picture 12—staining ISH III.

Diffuse staining was defined as continuous homogeneous nuclear staining, whereas punctate staining was defined as discrete dot-like nuclear signals. Focal distribution referred to localized signal presence limited to selected lesional areas, while dispersed distribution referred to widespread signal distribution throughout the lesion. The pictures of tissues derived from a cervix from different patients are presented in Figure 1.

### 2.6. Statistical Analysis

Analysis was conducted using the statistical software R (version 4.1.2). All analyses assumed a significance level of α = 0.05. Nominal variables were presented as n and %, and the median with quartiles 1 and 3 due to non-normal distributions. Normality of distribution was analyzed with a Shapiro–Wilk test and further verified with skewness and kurtosis. Comparisons were conducted with Pearson’s chi-square test or Fisher’s exact test, as appropriate. Diagnostic abilities of ISH II against HPV 6 or 11 and ISH III against HR HPV 16, 18, 31, 33, 35, 39, 45, 51, 52, 56, 58, and 66 were assessed with sensitivity, specificity, PPV (positive predictive value), NPV (negative predictive value), and accuracy. Sensitivity, specificity, PPV, NPV, and accuracy were calculated with corresponding 95% confidence intervals using the exact binomial method.

## 3. Results

Eighty-three women with a mean age of 33 were included in the analysis. Patient characteristics and the distribution of cytological and histopathological results are presented in Table 1. The staining types after using ISH II and ISH III probes, as well as the nature of the lesions, are also included in Table 1.

The stain may be described using the following features: diffuse, punctuate, and negative. As far as ISH II is concerned, there was no association with HPV genotypes 6 or 11 (*p* = 0.060). The distribution may be described as focal, dispersed, or negative. However, the distribution in ISH II was significantly associated with HPV genotype 6 or 11 (*p* < 0.001). The focal ISH II distribution was absent in HPV-positive cases (0/8) and was observed in 33.3% of HPV-negative cases (25/75). Dispersed distribution of ISH II was observed more frequently among patients with positive HPV compared to patients with negative HPV (62.5%, n = 5 vs. 2.7%, n = 2). Negative distribution of ISH II was observed less frequently among patients with positive HPV compared to patients with negative HPV (37.5%, n = 3 vs. 64.0%), Table 2.

Structure of stain in ISH III (diffused/punct/negative) was significantly associated with HPV outcome (genotypes 16, 18, 31, 33, 35, 39, 45, 51, 52, 56, 58, 66), *p* = 0.020. Among patients with positive HPV, 27.3% had a diffuse stain in ISH III, while no patient had a diffuse stain in ISH III among patients with negative HPV. Punct stain in ISH III was observed less frequently among patients with positive HPV compared to patients with negative HPV (59.1% vs. 76.5%). Negative stain in ISH III was observed less frequently among patients with positive HPV compared to patients with negative HPV (13.6%, n = 9 vs. 23.5%, n = 4). Structure of distribution in ISH III (focal/dispersed/negative) was not associated with HPV outcome, *p* = 0.145, as presented in Table 3.

Structure of ISH II stain was not associated with biopsy outcome (LSIL/HSIL), *p* = 0.166. Structure of ISH II distribution was not associated with biopsy outcome (LSIL/HSIL), *p* = 0.506. Structure of ISH III stain was not associated with biopsy outcome (LSIL/HSIL), *p* = 0.252. Structure of distribution in ISH III was significantly associated with biopsy outcome (LSIL/HSIL), *p* = 0.015. The focal distribution in ISH III was more frequent among patients with LSIL than among those with HSIL (44.1% vs. 25.0%). A dispersed distribution in ISH III was observed less frequently among patients with LSIL than among those with HSIL (32.4% vs. 64.6%). A negative distribution in ISH III was observed more frequently among patients with LSIL than among those with HSIL (23.5%, n = 8 vs. 10.4%, n = 5), as shown in Table 4.

A positive ISH II (stain/distribution) outcome predicted a positive HPV outcome (6 or 11) with a sensitivity of 62.50% (CI 95%: 24.49–91.48%) and a specificity of 64.00% (CI 95%: 52.09–74.77%). Overall accuracy was 63.86% (CI 95%: 52.57–74.12%), as presented in Table 5.

Positive outcome of ISH III (stain/distribution) predicted positive HPV outcome (any of 16, 18, 31, 33, 35, 39, 45, 51, 52, 56, 58, 66) with sensitivity of 86.36% (CI 95%: 75.69–93.57%) and specificity of 23.53% (CI 95%: 6.81–49.90%). Overall accuracy was 73.49% (CI 95%: 62.66–82.58%), as shown in Table 6.

## 4. Discussion

ISH is easy to perform and provides a reproducible method for detecting HPV on paraffin-embedded tissue sections. The advantage of this system over other methods that require the destruction of target cells, such as nucleic acid capture techniques or polymerase chain reaction (PCR), is its ability to test archival samples and to report HPV ISH results in the context of tissue morphology. Positive results aid in classifying normal and abnormal cells and tissues and complement conventional histopathological examinations. However, the interpretation of this product’s results should be performed by a qualified pathologist in conjunction with histological examination, relevant clinical information, and consultation with a clinician.

There is limited literature on the use of ISH in clinical practice, but the results reported by other authors are consistent with ours. In our cohort, ISH III achieved high sensitivity with low specificity. This pattern suggests that ISH III may be best positioned as a ‘rule-out’ adjunct when negative (i.e., a negative ISH III result makes HR-HPV less likely), whereas a positive result should be interpreted cautiously and preferably in combination with PCR genotyping, p16 immunohistochemistry, and morphological assessment. Potential contributors to low specificity might include the probe spectrum (limited genotype panel), low-copy or fragmented DNA in FFPE material, background staining, and the subjective threshold for calling weak punctate signals as positive; explicitly discussing these points helps understand clinical applicability.

Guo M. et al. reported that ISH and PCR demonstrated fair-to-good agreement in detecting HPV DNA across all CIN categories, with no significant differences overall. However, ISH detected significantly fewer HPV-positive cases in cancer specimens compared with PCR. Eleven cancer cases that were ISH-negative but PCR-positive tested positive for HPV types detectable by the Inform HPV III Probe. In five of these cases, significantly higher levels of integrated HPV16 were observed compared with ISH-positive cases [11]. These findings suggest that lower episomal HPV16 copy numbers in cancer may contribute to false-negative ISH results. The punctate HPV signal pattern increased significantly with cervical lesion grade. However, no significant difference in HPV16 integration status was observed between cases with only punctate signals and those with mixed punctate and diffuse signals. The authors concluded that ISH using the Inform HPV III Probe is comparable to PCR for detecting HPV DNA in cervical tissue with CIN. They proposed that false-negative ISH results may be related to low episomal HPV16 viral copy numbers rather than limitations in the probe’s ability to detect specific HPV types. Consistent with our findings, they also noted that HPV signal patterns—whether mixed, punctate, or diffuse—cannot reliably predict viral integration status. However, our conclusions apply specifically to precancerous cervical lesions (CIN) and the utility of ISH in invasive cervical cancer remains a separate question requiring dedicated investigation.

Researchers from Brazil explored the use of ISH probes to identify factors contributing to cervical cancer progression in cervical tissue. They utilized knowledge of HPV DNA integration in neoplastic transformation, the potential use of ISH probes, and the nature of the signal, in which a diffuse signal indicates episomal HPV and a punctate signal indicates integrated HPV. De Marchi Triglia R et al. aimed to verify if a punctate pattern could be a marker of CIN1 that progresses. The punctate signal was observed across all epithelial layers. The mean coefficient between the number of cells with punctate and diffuse signals was 3.5 times higher in the progression group, and this difference was statistically significant. The average percentage of nuclei with a punctate pattern in the basal region was 0.5% in cases without progression and 11% in cases with progression. However, in the superficial layer, no correlation with progression was observed. Punctate signals were associated with progression only in basal cells, thereby identifying CIN1 lesions with potentially aggressive behavior [12].

In a slightly older study by Unger ER et al., 165 archival paraffin blocks from patients with histopathologically diagnosed cervical cancer were evaluated. The results obtained were quite favorable for ISH and PCR, as a similar number of discordant results were ISH+/PCR− (23) and ISH−/PCR+ (18). However, the ISH−/PCR+ results can be partially explained by the absence of some HPVs in the ISH probe (eight cases). A limitation of this study is the lack of comparison of results across histopathological cases and the lack of assessment of stain structure and distribution in the reported cervical cancer cases [13].

In a study by Kalesidis T et al., PCR and ISH were studied to detect human papillomavirus (HPV) in cytologic and histologic specimens, respectively. ISH was a useful method for detection of HPV, even in a large fraction of samples with normal cytologic or biopsy findings. The authors suggested that when used together and evaluated in conjunction with histologic sections, ISH is a useful tool for ancillary molecular testing of HPV infection in cervical lesions, especially in CIN 2+ histological lesions where its analytic sensitivities and specificities were as good as those of PCR testing [14]. These results are in line with ours that a positive ISH II outcome predicted a positive HPV outcome (6 or 11) with a sensitivity of 62.50% and a specificity of 64.00%, and positive outcome of ISH III predicted positive HPV outcome (any of 16, 18, 31, 33, 35, 39, 45, 51, 52, 56, 58, 66) with sensitivity of 86.36% and specificity of 23.53%.

Mills AM et al. also evaluated ISH performance in tissues beyond the cervix. Given that HPV is implicated in head and neck as well as anogenital cancers, they compared high-risk HPV RNA ISH (HR-RISH) with DNA PCR, p16 immunohistochemistry, and a previously available HPV DNA ISH assay in HPV-related anogenital and head and neck neoplasms. HR-RISH demonstrated a sensitivity of ≥97% for PCR-positive and p16-positive neoplasia, as well as for morphologically defined anogenital high-grade squamous intraepithelial lesions and invasive squamous cell carcinomas. It was also positive in 78% of anogenital low-grade squamous intraepithelial lesions, including 81% of CIN1 cases. Notably, a subset of lesions that were PCR-negative or invalid and p16-negative showed positive HR-RISH results. These findings indicate that HR-RISH is a robust method for detecting HR-HPV-related neoplasia and offer additional insight into HPV pathobiology [15]. It should be emphasized that the study by Mills et al. evaluated HR-HPV RNA ISH, whereas the present study used DNA ISH. These techniques differ biologically and diagnostically, as RNA ISH detects transcriptionally active viral infection, while DNA ISH identifies the presence of viral genetic material regardless of transcriptional activity. Therefore, direct comparison between these methods should be interpreted cautiously. Nevertheless, due to the limited number of studies assessing ISH-based HPV detection in tissue specimens, we considered these data useful for broader methodological context.

A limitation of the present study is that ISH performed on FFPE tissue was compared with HPV PCR results obtained from liquid-based cytology samples rather than PCR performed on the same tissue block. Although this reflects routine clinical practice, direct molecular comparison within the same specimen could provide a more precise assessment of analytical concordance. The relatively wide confidence intervals observed in several diagnostic accuracy estimates reflect the limited cohort size and subgroup imbalance, thereby reducing the precision of these estimates. An additional limitation of the study is the absence of fully blinded independent assessment by two pathologists and the lack of formal interobserver agreement analysis. Although the histopathological interpretation was ultimately performed by an experienced pathologist, the absence of independent blinded reassessment and reproducibility metrics may limit the methodological robustness of manual ISH signal evaluation.

Overall, available research suggests that ISH is a reliable and useful method for detecting HPV in tissue samples, yielding results comparable to PCR in many situations. Despite certain limitations related to viral load and signal interpretation, ISH remains a complementary approach to histopathological evaluation, particularly when molecular results must be interpreted in the context of tissue morphology. PCR-based HPV testing remains the preferred diagnostic approach in routine clinical practice; however, ISH may provide complementary value in selected situations, particularly when archival FFPE tissue is the only available material or when direct morphological localization of HPV within lesional tissue is clinically relevant. It should be emphasized that the number of studies and analyzed cases is small, leaving room for improvement in clinical practice, and these cases are primarily focused on research and laboratory diagnostics.

## 5. Conclusions

In conclusion, ISH may serve as a complementary tool for HPV detection in archival FFPE cervical tissue, particularly when preservation of tissue morphology and spatial localization of viral signals are clinically relevant. Although PCR-based testing remains the preferred diagnostic method due to its higher analytical performance and less invasive sampling, ISH may provide additional value in morphology-oriented assessment and retrospective tissue analysis. Therefore, ISH should be considered an adjunctive rather than replacement method for HPV diagnostics.

## Figures and Tables

**Table 1:** Group characteristics.

Characteristics	Values
N	83
Age, years, median (Q1; Q3)	33.00 (30.00; 37.50)
ISH II Stain, n (%)	
Diffused	1 (1.2)
Punct	31 (37.3)
Negative	51 (61.4)
ISH II Distribution, n (%)	
Focal	25 (30.1)
Dispersed	7 (8.4)
Negative	51 (61.4)
ISH III Stain, n (%)	
Diffused	18 (21.7)
Punct	52 (62.7)
Negative	13 (15.7)
ISH III Distribution, n (%)	
Focal	28 (33.7)
Dispersed	42 (50.6)
Negative	13 (15.7)
Cytology, n (%)	
NILM	5 (6.0)
ASC-US	14 (16.9)
LSIL	37 (44.6)
ASC-H	14 (16.9)
HSIL	13 (15.7)
Biopsy, n (%)	
NILM	1 (1.2)
LSIL	34 (41.0)
HSIL	48 (57.8)
HPV, n (%)	
Positive	73 (88.0)
Negative	10 (12.0)
HPV Genotype 6 or 11, n (%)	8 (9.6)
HPV Genotype any of: 16, 18, 31, 33, 35, 39, 45, 51, 52, 56, 58, 66, n (%)	66 (79.5)

Q1—first quartile, Q3—third quartile, n—number, ASC-US—Atypical Squamous Cells of Undetermined Significance, ASC-H—Atypical Squamous Cells, cannot exclude high-grade squamous intraepithelial lesion, LSIL—low-grade squamous intraepithelial lesion, HSIL—high-grade squamous intraepithelial lesion, NILM—Negative for Intraepithelial Lesion or Malignancy, HPV—human papillomavirus, *p*—*p*-value.

**Table 2:** ISH II stain and ISH II distribution against HPV genotype 6 or 11.

Variables	HPV Genotype 6 or 11		*p*
	Positive (n = 8)	Negative (n = 75)	
ISH II Stain, n (%)			
Diffused	1 (12.5)	0 (0.0)	0.060
Punct	4 (50.0)	27 (36.0)	
Negative	3 (37.5)	48 (64.0)	
ISH II Distribution, n (%)			
Focal	0 (0.0)	25 (33.3)	<0.001
Dispersed	5 (62.5)	2 (2.7)	
Negative	3 (37.5)	48 (64.0)	

n—number, *p*—*p*-value. Comparisons were made with Fisher’s exact test.

**Table 3:** ISH III stain and ISH III distribution against HPV outcome.

Variables	HPV Genotype Any of 16, 18, 31, 33, 35, 39, 45, 51, 52, 56, 58, 66		*p*
	Positive (n = 66)	Negative (n = 17)	
ISH III Stain, n (%)			
Diffused	18 (27.3)	0 (0.0)	0.020
Punct	39 (59.1)	13 (76.5)	
Negative	9 (13.6)	4 (23.5)	
ISH III Distribution, n (%)			
Focal	20 (30.3)	8 (47.1)	0.145
Dispersed	37 (56.1)	5 (29.4)	
Negative	9 (13.6)	4 (23.5)	

n—number, *p*—*p*-value. Comparisons were made with Fisher’s exact test.

**Table 4:** ISH II stain and ISH II distribution against biopsy outcome.

Variables	Biopsy			*p*
	NILM (n = 1)	LSIL (n = 34)	HSIL (n = 48)	
ISH II Stain, n (%)				
Diffused	0 (0.0)	1 (2.9)	0 (0.0)	0.166 ^1^
Punct	0 (0.0)	10 (29.4)	21 (43.8)	
Negative	1 (100.0)	23 (67.6)	27 (56.2)	
ISH II Distribution, n (%)				
Focal	0 (0.0)	8 (23.5)	17 (35.4)	0.506 ^1^
Dispersed	0 (0.0)	3 (8.8)	4 (8.3)	
Negative	1 (100.0)	23 (67.6)	27 (56.2)	
ISH III Stain, n (%)				
Diffused	0 (0.0)	6 (17.6)	12 (25.0)	0.252
Punct	1 (100.0)	20 (58.8)	31 (64.6)	
Negative	0 (0.0)	8 (23.5)	5 (10.4)	
ISH III Distribution, n (%)				
Focal	1 (100.0)	15 (44.1)	12 (25.0)	0.015
Dispersed	0 (0.0)	11 (32.4)	31 (64.6)	
Negative	0 (0.0)	8 (23.5)	5 (10.4)	

n—number, NILM—Negative for Intraepithelial Lesion or Malignancy, LSIL—low-grade squamous intraepithelial lesion, HSIL—high-grade squamous intraepithelial lesion, *p*—outcome of comparisons between LSIL and HSIL, made with Pearson’s chi-square test or Fisher’s exact test ^1^, as appropriate.

**Table 5:** Sensitivity and specificity of ISH II stain and ISH II distribution in HPV 6 or 11.

Variable		HPV 6 or 11			Sensitivity, %	Specificity, %	PPV, %	NPV, %	Accuracy, %
		Yes, n = 8	No, n = 75	Total, n = 83					
ISH II *	+	5	27	32	62.50 (24.49–91.48)	64.00 (52.09–74.77)	15.62 (9.09–25.53)	94.12 (86.55–97.55)	63.86 (52.57–74.12)
	−	3	48	51					

PPV—positive predictive value, NPV—negative predictive value, n—number. * Positive outcome defined as positive ISH II stain (diffused + punct)/positive ISH II distribution (focal + dispersed).

**Table 6:** Sensitivity and specificity of ISH III stain and ISH III distribution in HPV genotype any of 16, 18, 31, 33, 35, 39, 45, 51, 52, 56, 58, 66.

Variable		HPV 16, 18, 31, 33, 35, 39, 45, 51, 52, 56, 58, 66			Sensitivity, %	Specificity, %	PPV, %	NPV, %	Accuracy, %
		Yes, n = 66	No, n = 17	Total, n = 83					
ISH III *	+	57	13	70	86.36 (75.69–93.57)	23.53 (6.81–49.90)	81.43 (76.81–85.30)	30.77 (13.46–55.96)	73.49 (62.66–82.58)
	−	9	4	13					

PPV—positive predictive value, NPV—negative predictive value, n—number. * Positive outcome defined as positive ISH II stain (diffused + punct)/positive ISH II distribution (focal + dispersed).

## Data Availability

All source data are available from the corresponding author.
